# Nano-selenium strengthens potato resistance to potato scab induced by *Streptomyces* spp., increases yield, and elevates tuber quality by influencing rhizosphere microbiomes

**DOI:** 10.3389/fpls.2025.1523174

**Published:** 2025-02-03

**Authors:** Haixu Liu, Yan Zhang, Lili Zhang, Yingjie Liu, Yufei Chen, Ying Shi

**Affiliations:** ^1^ College of Agriculture, Northeast Agricultural University, Harbin, China; ^2^ Food and Cash Crops Branch, Harbin Academy of Agricultural Sciences, Harbin, China

**Keywords:** nano-selenium, rhizosphere bacterial microbiome, tuber quality, yield, antioxidant enzyme

## Abstract

**Introduction:**

The application of selenium could directly or indirectly modulate the activity of antioxidant enzymes in crops, thereby mitigating the detrimental effects of abiotic and biotic stresses on crop health. However, there are few studies on the effects of nano-selenium fertilizer on potato scab caused by *Streptomyces* spp., potato yield and tuber quality.

**Methods:**

We aimed to elucidate the impact of nano-selenium fertilizer on potato disease resistance, yield, tuber quality, antioxidant enzyme activity and rhizosphere soil bacterial communities, and to determine the optimal frequency and growth stages of nano-selenium fertilizer spraying.

**Results and discussion:**

The application of nano-selenium fertilizer twice during the seedling stage significantly reduced the disease index of potato scab, enhanced potato yield, tuber quality (dry matter, Vitamin C, crude protein, and selenium content), and antioxidant enzyme activity (glutathione peroxidase, peroxidase, polyphenol oxidase, superoxide dismutase, and phenylalanine ammonia lyase). The diversity of the rhizosphere bacterial community of potatoes subjected to selenium fertilizer spraying at the seedling stage increased significantly, and concurrently, the symbiotic network of rhizosphere bacterial microbiome grew more complex. Beneficial microorganisms such as bacteria of the genus *Bacillus* were enriched in the rhizosphere soil. The current study provided theoretical support for the exploration of a potato selenium-enriched technology system and supplies scientific guidance for the utilization of nano-selenium.

## 1 Introduction

Selenium is crucial for maintaining normal physiological functions in the human body. It actively participates in regulating various physiological activities and plays a vital role in enhancing immune function, promoting antioxidation, delaying aging, and inhibiting tumor growth (Farooq et al., 2019; Rayman, 2012). The human body requires a daily dietary intake of approximately 200 μg of selenium to ensure optimal health (Wu et al., 2015). Selenium cannot be synthesized autonomously in the human body and must be obtained from dietary foods such as grains to obtain the necessary selenium for humans. Consuming agricultural products rich in selenium was an effective method for individuals with selenium deficiency to supplement their selenium intake. China holds the title of the global leader in potato consumption (Birch et al., 2012). Potatoes are the fourth major staple crop, with a cultivation area smaller than that of wheat, rice, and maize. They possess advantageous traits such as a short growth cycle, with a rich nutritional profile comprising starch, protein, sugars, and Vitamin C (Vc). However, potatoes belong to non-selenium-accumulating plants, and their absorption and accumulation of selenium are influenced by both endogenous genetic factors and exogenous environmental conditions. Therefore, the utilization of selenium biofortification for cultivating selenium-enriched potatoes holds significant implications for human health and is currently a research focus (Somalraju et al., 2021; Zhang et al., 2019).

The commonly utilized selenium fertilizers primarily encompassed inorganic selenium, organic selenium, and nano-selenium (Dinh et al., 2018). The particle size of nano-selenium is usually at the nanometer level and has a large specific surface area, which makes it easier to contact and be absorbed by the surfaces of crop roots or leaves (Wang et al., 2020). Inorganic selenium, such as sodium selenite, has a relatively narrow range between the effective dose and the toxic dose. Excessive use can easily cause crop poisoning, have an inhibitory effect on plant growth, and even cause plant death (Ramos et al., 2011). Due to its special nano-structure and chemical properties, its toxicity is relatively low, which is safer for the growth and development of crops (Zhang et al., 2023b). Inorganic selenium is prone to chemical reactions in the soil and may have certain impacts on the soil ecological environment, such as changing the structure of the soil microbial community. However, the stability of nano-selenium is relatively good. Its activity and mobility in the soil are relatively low, and it has a smaller impact on the soil environment. Furthermore, nano-selenium is also safer for soil microorganisms. It has been reported that SeO_3_^2-^ can be easily transported across the membrane via binding to the sulfhydryl sites on the cell membrane or by phosphate transfer protein. In contrast, nano-selenium is in a reduced state. The Se atom is coated with a layer of extracellular polymer, making it difficult to enter the microorganism directly. It is more in line with the requirements of sustainable agricultural development (Liu et al., 2021). The absorption of selenium by different crops varies significantly during the process of enhancing crop selenium nutrition. Therefore, reasonable selection of spraying frequency and growth stage is also an effective strategy for increasing the selenium content in crops.

The application of selenium not only enhanced the selenium content in potato tubers, but also facilitated an increase in yield and improvement in quality (Zhang et al., 2019). The application of sodium selenate significantly increased the yield, crude protein and Vc content of potato tubers, while there was no statistically significant difference in the starch content of tubers (Yin et al., 2015). The literature has reported the application of nano-selenium fertilizer to enhance the quality of tea and celery (Li et al., 2020; Xiangchun et al., 2024), but there is a lack of research on its application for improving potato tuber quality.

Potato scab caused by *Streptomyces* spp. is a soil-borne disease that seriously affects potato yield. The annual occurrence of potato scab led to significant global economic losses (Hiltunen et al., 2016). The potato scab resulted in the formation of scabby, raised, or sunken lesions on the surface of tubers, which significantly impacted the quality of commercial potatoes and made them susceptible to storage issues (Braun et al., 2017). Currently, chemical control remained the primary approach for controlling potato scab. However, prolonged use of pesticides led to pathogens developing resistance, with potential implications for soil contamination through pesticide residues that might subsequently impact the quality and safety of agricultural products (Nomen et al., 2012). Selenium has a direct inhibitory effect on the growth of plant pathogens. Under *in vitro* conditions, inorganic selenium can inhibit the growth of *Phanerochaete chrysosporium* (Espinosa-Ortiz et al., 2015) and *Fusarium graminearum* (Mao et al., 2020). Furthermore, the application of selenium could directly or indirectly modulate the activity of antioxidant enzymes in crops, thereby mitigating the detrimental effects of abiotic (heavy metal, drought) and biotic (*Fusarium graminearum*, and *Sclerotinia sclerotiorum*) stresses on crop health (Handa et al., 2018; Rady et al., 2020; Somalraju et al., 2021).

The rhizosphere soil microorganisms play a crucial role in the growth and development of crops. A healthy soil microbial community is imperative for optimal plant growth and enhancing disease resistance (Wang et al., 2023). The presence of selenium also has an influence on the composition and resilience of the rhizosphere soil microbial community. The application of selenium treatment enhanced the growth of *Atractylodes macrocephala* by increasing the relative abundance of *Burkholderia* and *Cupriavidus* in the rhizosphere soil (Zhou et al., 2021). Nano-selenium treatment increased the abundance of beneficial rhizosphere microorganisms (Bacteria of the genus *Pseudomonas* and *Bacillus*) in the rhizosphere soil, thereby improving the nutrient utilization rate of plants (Chuanxi et al., 2021). In addition to modulating nutrient uptake by plants via microorganisms, selenium can also confer tolerance to environmental stress through microbial communities. The soil selenium might increase the microbiome diversity and the relative abundance of *Rhizobium*, *Flavobacterium*, and other beneficial bacteria in the rhizosphere thus inhibiting the occurrence of Sclerotinia stem rot (Liu et al., 2019). However, the potential effects of nano-selenium on potato scab, yield and tuber quality, and the regulation of rhizosphere microbial community remain unclear.

The objective of this study is (1) to determine the optimal spraying frequencies and growth stages for potato application of nano-selenium fertilizer; (2) to elucidate the impact of nano-selenium fertilizer on potato scab resistance, yield, tuber quality, antioxidant enzyme activity, and rhizosphere soil bacterial community; (3) to investigate the relationship between changes in rhizosphere soil bacterial community and potato scab resistance, yield, and tuber quality. The findings of this research provide theoretical support for the development of a potato selenium-enriched technology system and offer scientific guidance on the utilization of nano-selenium.

## 2 Materials and methods

### 2.1 Experimental materials and design

The potato variety Dongnong 310 was obtained from the Potato Research Institute of Northeast Agricultural University in Heilongjiang Province, China. The nano-selenium (Se0 nano-particles with a main size of 50 - 78 nm) containing bioactive selenium at a concentration of 1500 mg/L was provided by Guilin Jiqi Biochemical Co., Ltd. This research was conducted in the Xiangyang experimental demonstration base (N: 45.775837; E: 126.914749) of Northeast Agricultural University in Harbin, Heilongjiang Province, China. The area had a temperate monsoon climate and an average annual precipitation of 500 - 600 mm. The soil type was chernozem (organic matter 33.69 g·kg^-1^, alkali hydrolyzable nitrogen 114.42 mg·kg^-1^, available phosphorus 18.25 mg·kg^-1^, available potassium 136.28 mg·kg^-1^, pH 6.2, soil selenium 0.129 mg·kg^-1^). The fertilizer application rates were as follows: urea (N 46%) at a rate of 75 kg·ha^-1^, diammonium phosphate (N 18%, P_2_O_5_ 46%) at a rate of 150 kg/ha, and potassium sulfate (K_2_O 50%) at a rate of 75 kg·ha^-1^.

The experimental design adopted a randomized order for each treatment with three replicates. In the experiment, each plot had a ridge width of 0.8 m, a ridge length of 5 m, and consisted of 4 ridges with a plant spacing of 0.2 m, and a plot area of 16 m^2^. The experimental periods were as follows: planting on May 9th, 2022, and harvesting on September 26th, 2022; planting on May 2nd, 2023, and harvesting on September 28th, 2023.

In the 2022 experiment, at the potato seedling stage (June 20th), the nano-selenium solution (1500 mg·L^-1^) was diluted 300 times with tap water for foliar spraying treatment. The treatments of spraying once, spraying twice, and spraying three times were respectively set up, with a spraying interval of seven days. The application concentration was maintained at 5 mg·L^-1^, with an application volume of 4500 mL·ha^-1^ (Li et al., 2020). The control group was not sprayed with nano-selenium fertilizer. Based on the effects of different spraying frequency treatments on the quality of potato tubers, the spraying frequency for the second-year experiment was determined. In the 2023 experiment, the application of nano-selenium through foliar spraying at different growth stages of potatoes, namely the seedling stage (June 10), tuber initiation stage (July 1), tuber bulking stage (August 1), and tuber maturity stage (August 25). A second round of spraying was performed seven days after the initial application. In contrast, the control group was not sprayed with nano-selenium fertilizer.

### 2.2 Investigation of potato scab

The experimental site was selected in the field where potato scab occurred naturally. At harvest, the potato yields of each treatment were determined. By observing the diseased potatoes that showed typical symptoms of potato scab, including the formation of suberized scab-like lesions on the surface of tubers, which had irregular shapes, different sizes and were usually brown to black in color. To reflect the disease occurrence degree of potato scab more comprehensively and quantitatively, the disease index was determined according to the established classification standard. The classification standard of potato scab is as follows (Cui et al., 2020): Grade 0 represents healthy potatoes without any disease spots on their skin; Grade 1 indicates the presence of 1-2 diseased spots on the surface of potato skin, with a total area not exceeding 1/4 of the skin’s surface area; Grade 2 signifies the existence of 3-5 lesions on the potato skin, covering an area between 1/4 and 1/3 of its surface; Grade 3 denotes the occurrence of 5-10 lesions on the potato skin, occupying an area ranging from 1/3 to half of its surface; and finally, Grade 4 represents more than ten disease spots on the potato skin, with a total area surpassing half of its surface. The Disease index was calculated by referring to the following Formula 1:


(1) $$\text{Disease index}=\sum^{}\frac{\text{number of diseased plants at relativel levels }\times \text{ relative level}}{\text{number of investigated plants }\times \text{ maximum incidence level}}\times 100$$

### 2.3 Determination of tuber quality

Dry matter content was determined using the Drying method (Islam et al., 2022); Starch content was determined using iodine colorimetry (Wang et al., 2019); Vc content was determined using 2,6-dichloroindophenol titration (Queb-González et al., 2020); Crude protein content was determined using near infrared reflectance spectroscopy (Schum and Jansen, 2012); Tuber selenium content was determined as follows (Lin et al., 2012): The tuber samples were ground and digested in a mixture of HNO_3_ and HClO_4_ (4:1). The selenium content in the tubers was determined using inductively coupled plasma atomic emission spectrometry (ICP-AES) (Agilent 7800, USA).

Potato tuber contains a variety of nutritional indicators for evaluating the quality, so the fuzzy mathematics membership function method was used to evaluate the differences of the main nutritional indicators in potato tuber under different treatments (Chen et al., 2012). First, calculate the membership function value of a single quality index in potato tuber, and then calculate the average membership function value of quality (MFVQ) index according to the membership function value of a single quality index. The higher the MFVQ, the better the comprehensive quality. The following formula is applied:


(2) $${\text{X}}_{\text{i}(\text{μ})}=\frac{\text{X}-{\text{X}}_{\text{min}}}{{\text{X}}_{\text{max}}-{\text{X}}_{\text{min}}}$$

(3) $$\text{MFVQ}=\frac{{\text{X}}_{1(\text{μ})}+{\text{X}}_{2(\text{μ})}\cdots {\text{X}}_{\text{n(μ})}}{\text{n}}$$

Xi_(μ)_ is the average subordinate value of a single quality index in a process, X is the value of the single quality index result, x_min_ is the minimum value of the single quality index result in the process, and x_max_ is the maximum value of the single quality index result in the process. X_1(μ)_, X_2(μ)_,⋯X_n(μ)_, represent the membership function value of a single quality index, and n is the number of quality indexes.

### 2.4 Determination of antioxidant enzyme activity in tubers

Weigh approximately 0.1g of the tuber tissue, add 1 mL of the extraction solution for homogenization in an ice bath. Centrifuge at 10,000g and 4°C for 10 minutes, take the supernatant and place it on ice for detection. According to the requirements of the kit (adsbio, China), the absorbance was determined using a spectrophotometer, and the activities of glutathione peroxidase (GSH-Px), peroxidase (POD), polyphenol oxidase (PPO), superoxide dismutase (SOD), and phenylalanine ammonia-lyase (PAL) of each sample were calculated as per the instructions.

### 2.5 Determination of rhizosphere bacterial microbiome

The potato plants were uprooted seven days before harvest, gently shook off loose soil around the roots and removed bulk soil. The rhizosphere soil from each treatment group was collected and promptly preserved in liquid nitrogen. Three samples were collected for each treatment group. The rhizosphere soil DNA was extracted using the DiFast DNA Spin Kit (Dining, China). The bacterial V4 region was amplified utilizing primers 515F (GTGCCAGCMGCCGCGGTAA) and 806R (GGACTACHVGGGTWTCTAAT) (Zhang et al., 2023a). The PCR products were subjected to sequencing using the Illumina NovaSeq platform provided by Pearsonalbio (Shanghai, China). The vsearch method was used for primer removal, splicing, quality filtering, duplicate removal, mosaic detection, and clustering (Rognes et al., 2016). The 16S rRNA gene was annotated using Silva database (http://greengenes.secondgenome.com/) (Quast et al., 2013).

### 2.6 Statistical analysis

QIIME 2 was used to process the bacterial 16S rRNA gene sequences (Bolyen et al., 2019). Permutational multivariate analysis of variance (PERMANOVA, with transformed data by Bray-Curtis, permutation = 999) was carried out using the “vegan” package to explore the differences in the composition of rhizosphere bacterial communities. The co-occurrence network was visualized using Gephi v0.9.2 (Bastian et al., 2009). The biomarker bacteria were identified using Lefse (logarithmic LDA score>2.5, P< 0.05) (Xia et al., 2024). Spearman correlations between biomarker bacteria and tuber quality, disease index of potato scab, yield, and tuber antioxidant enzyme activity were calculated in SPSS 20.0 software (IBM SPSS Statistics, IBM Corporation, Armonk, NY, USA) and plotted as heatmaps in R (*P< 0.05, **P< 0.01). The plots were created using the “ggplot2” package in R (v4.2.3). The experimental data were compared with the mean values using Duncan’s Multiple Range Test in SPSS 20.0 software at a significance level of 5% (P< 0.05).

## 3 Results

### 3.1 Effect of spraying frequency of nano-selenium on potato

#### 3.1.1 Disease index of potato scab and potato yield

**Figure 1:**
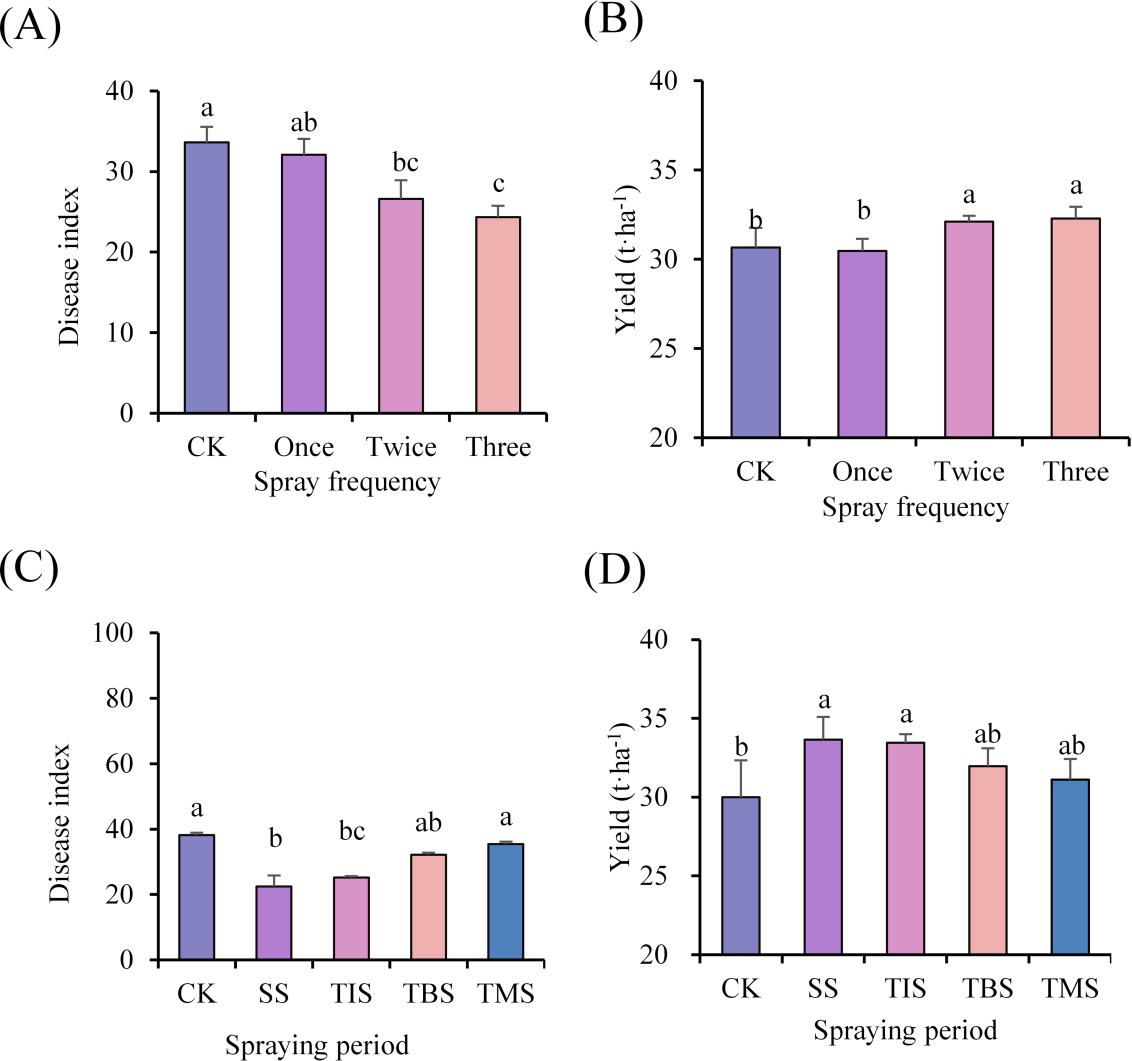
Effect of frequency of spraying nano-selenium on disease index of potato scab **(A)** and yield **(B)**. Effect of spraying nano-selenium twice at different growth stages on the disease index of potato scab **(C)** and the yield **(D)**. SS, TIS, TBS and TMS were seedling stage, tuber initiation stage, tuber bulking stage and tuber maturity stage, respectively. Different lowercase letters indicate significant differences between different treatments (P< 0.05).

The disease index of potato scab (24.34; CK 33.62) in the treatment of spraying three times was significantly lower than that of the CK, but there was no significant difference compared to the treatment of spraying twice. In the spraying twice and three times treatment, the potato yield was 32.10 and 32.29 t·ha^-1^, respectively, which was significantly higher than the yield from the CK at 30.67 t·ha^-1^, resulting in an increase of 4.66% and 5.28%, respectively. However, no significant difference was observed between the spraying twice and three times treatments (**Figures 1A, B**).

#### 3.1.2 Tuber quality

**Figure 2:**
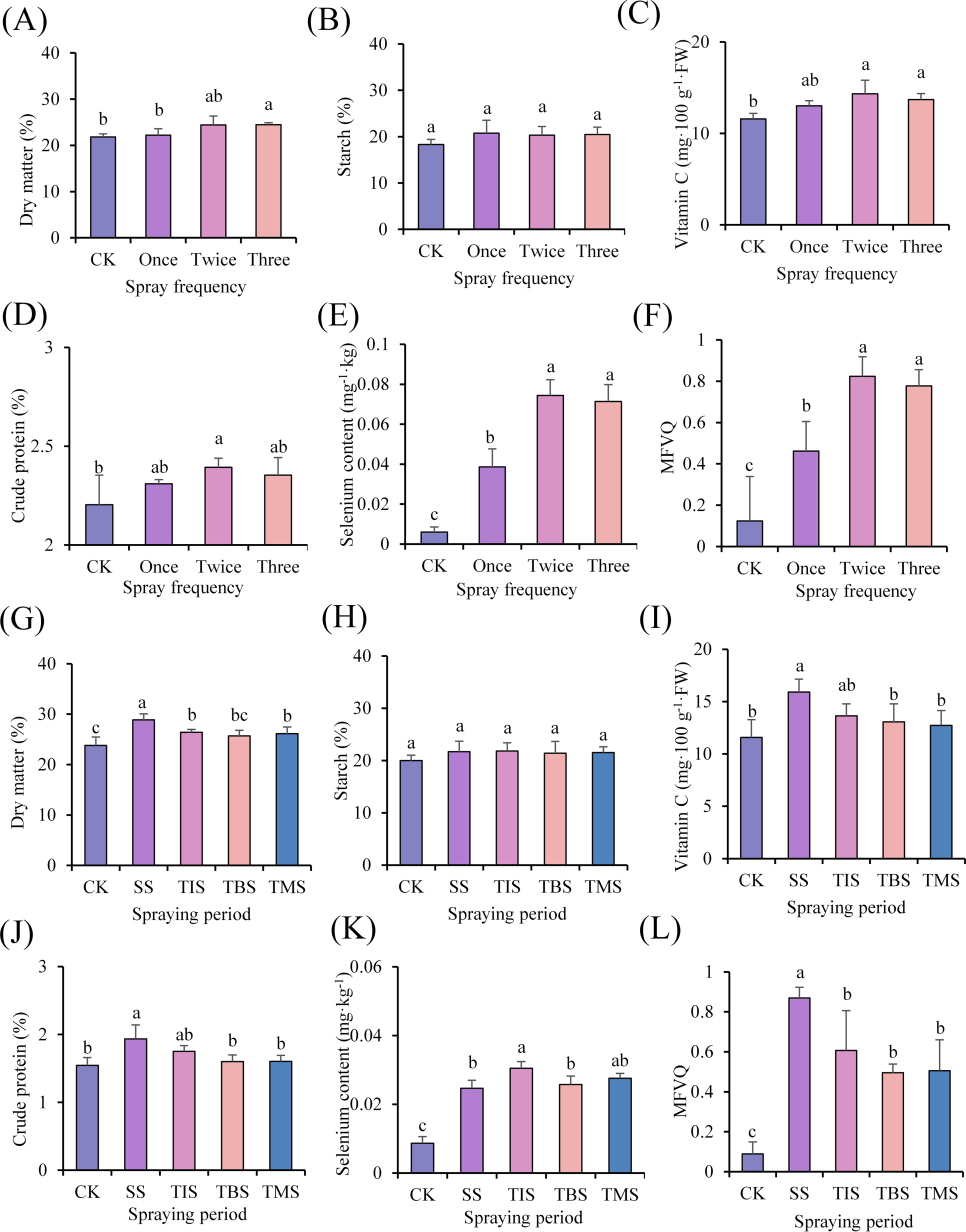
Effect of frequency of spraying nano-selenium on potato tuber quality **(A-F)**. Effect of spraying nano-selenium twice at different growth stages on the quality of potato tubers **(G-L)**. SS, TIS, TBS and TMS were seedling stage, tuber initiation stage, tuber bulking stage and tuber maturity stage, respectively. Different lowercase letters indicate significant differences between different treatments (P< 0.05).

In the spraying three times treatment, the dry matter content (24.46%; CK 21.85%) showed significant increases compared to the CK. In the spraying twice treatment, the Vc content (14.33 mg·100 g^-1^ FW; CK 11.58 mg·100 g^-1^ FW), crude protein content (2.39%; CK 2.20%) dry matter content (24.46%; CK 21.85%), and selenium content (0.074 mg·kg^-1^; CK 0.006 mg·kg^-1^) showed significant increases compared to the CK (**Figures 2A–E**). The tuber quality of different treatments was comprehensively compared, and in the spraying twice treatment, the MFVQ index of potato tuber was the highest (**Figure 2F**).

#### 3.1.3 Tuber antioxidant enzyme activity

**Figure 3:**
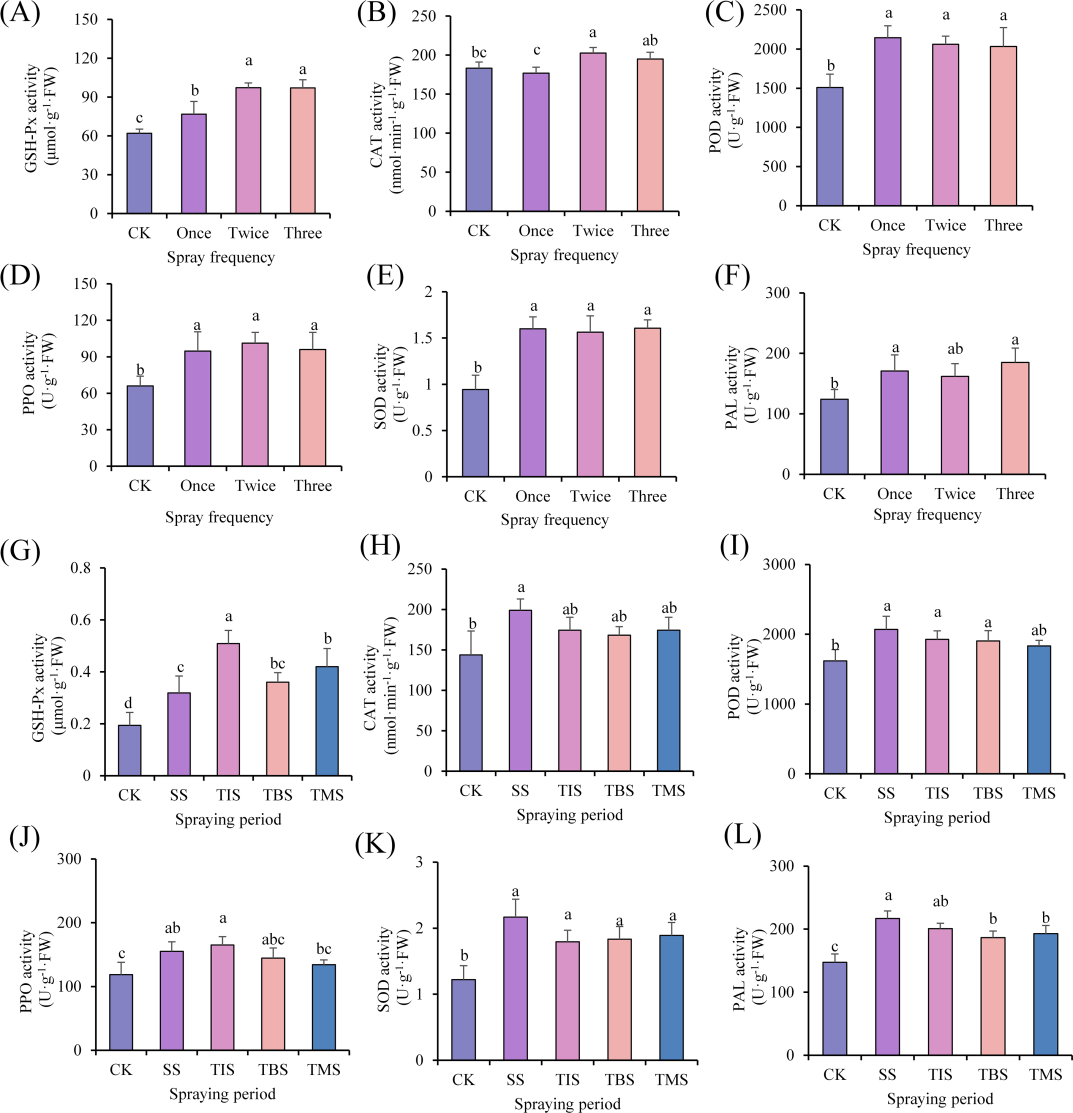
Effect of frequency of spraying nano-selenium on antioxidant enzyme activity of potato tuber **(A-F)**. Effect of spraying nano-selenium twice at different growth stages on antioxidant enzyme activities of potato tubers **(G-L)**. SS, TIS, TBS and TMS were seedling stage, tuber initiation stage, tuber bulking stage and tuber maturity stage, respectively. Different lowercase letters indicate significant differences between different treatments (P< 0.05).

The activities of GSH-Px, POD, PPO, SOD, and PAL in the tubers were significantly higher than those of CK in the spraying nano-selenium treatment. The CAT enzyme activity (202.61 nmol·min^-1^·g^-1^·FW; CK 183.28 nmol·min^-1^·g^-1^·FW) was significantly higher than that of CK in the spraying twice treatment. There was no significant difference in CAT enzyme activity between spraying once treatment and three times treatment and CK (**Figures 3A–F**). Based on the above effects of spraying nano-selenium frequency on potatoes, the final choice was to spray nano-selenium twice for the next experiment.

### 3.2 Effect of spraying nano-selenium at different stages on potato

#### 3.2.1 Disease index of potato scab and potato yield

The disease index of potato scab at the seedling stage treatment and tuber bulking stage treatment was significantly lower than that of the CK, with the disease index of potato scab at the seedling stage treatment being the lowest (22.41; CK 38.16). Furthermore, both potato yield at the seedling stage treatment and tuber initiation stage treatment were significantly higher than that of CK, with the highest yield observed at the seedling stage treatment (33.65 t·ha^-1^; CK 30.00 t·ha^-1^), representing a remarkable increase of 12.17% (**Figures 1C, D**).

#### 3.2.2 Tuber quality

In the seedling stage treatment, tuber initiation stage treatment, and tuber maturity stage treatment, dry matter content exhibited a significantly higher level compared to that of the CK. Moreover, the highest dry matter content was observed in tubers during the seedling stage treatment (28.88%; CK 23.81%). The application of nano-selenium did not yield a significant impact on the starch content in tubers. The Vc (15.90 mg·100 g^-1^·FW; CK 11.57 mg·100 g^-1^·FW) and crude protein content (1.93%; CK 1.54%) in the seedling stage treatment exhibited significantly higher levels compared to those in the CK, whereas no significant differences were observed in Vc and crude protein contents during treatments at other stages. The selenium content of potato tubers at each stage of treatment was significantly elevated compared to that of the CK, with the highest concentration recorded as 0.030 mg·kg^-1^ during the tuber initiation stage treatment (**Figures 2G–K**). The tuber quality of different treatments was comprehensively compared, and in the seedling stage treatment, the MFVQ index of potato tubers was the highest (**Figure 2L**).

#### 3.2.3 Tuber antioxidant enzyme activity

The activities of GSH-Px, SOD, and PAL were significantly higher than those of CK in the treatment of spraying nano-selenium. Specifically, the activities of GSH-Px (0.32 μmol·g^-1^·FW; CK 0.19 μmol·g^-1^·FW), SOD (2.17 U·g^-1^·FW; CK 1.22 U·g^-1^·FW), and PAL (216.72 U·g^-1^·FW; CK 147.48 U·g^-1^·FW) were the highest in the seedling stage. The CAT activity at the seedling stage (198.90 nmol·min^-1^·g-1·FW; CK 143.82 nmol·min^-1^·g^-1^·FW) was significantly higher than that of the CK. There were no significant differences in CAT activity between CK and the treatment groups at other stages. The POD activity of the seedling stage treatment, tuber initiation stage treatment, and tuber bulking stage treatment was significantly higher than that of CK. Among these stages, the highest POD activity was observed at the seedling stage (2067.57 U·g^-1^·FW; CK 1616.81 U·g^-1^·FW). Additionally, both treatments showed significantly higher PPO activities compared to CK at both the seedling stage and tuber initiation stage, with no significant difference between them (**Figures 3G–L**).

### 3.3 Effects of spraying nano-selenium at different growth stages on rhizosphere bacterial microbiome

**Figure 4:**
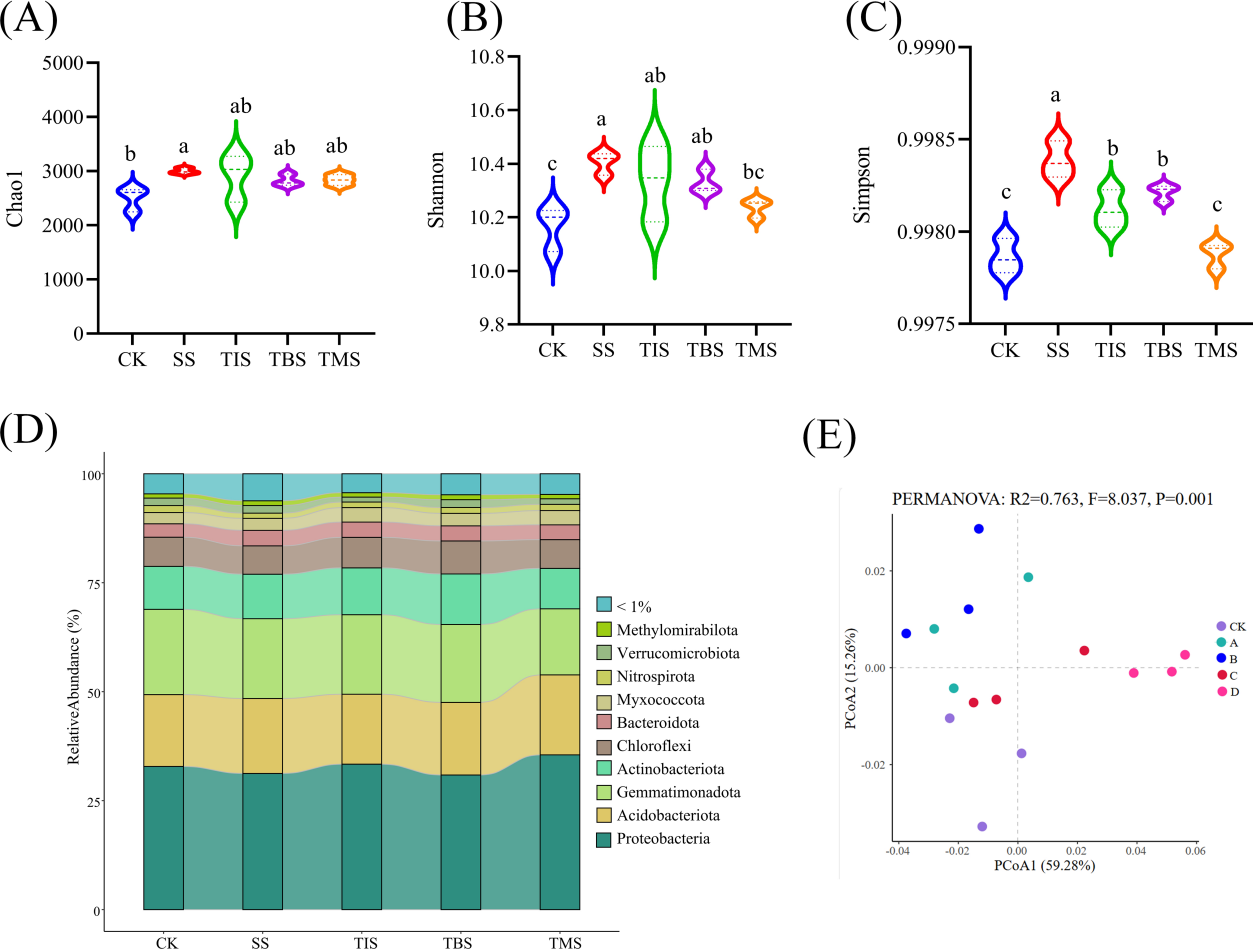
Effect of spraying nano-selenium twice at different growth stages on rhizosphere microbial community. The rhizosphere bacterial α-diversity indices of different treatment groups include Chao1 **(A)**, Shannon **(B)** and Simpson **(C)**. Different lowercase letters indicate significant differences between different treatments (P< 0.05). The species abundance at the phylum level of rhizosphere microorganisms in different treatment groups **(D)**. PCoA analysis of rhizosphere bacterial β diversity in different treatment groups **(E)**. SS, TIS, TBS and TMS were seedling stage, tuber initiation stage, tuber bulking stage and tuber maturity stage, respectively.

The application of nano-selenium at different growth stages had an impact on the microbial diversity and the Alpha diversity of potato rhizosphere soil. Specifically, the seedling stage treatment exhibited the highest Chao1, Shannon, and Simpson index of rhizosphere microorganisms (**Figures 4A–C**). The analysis of dominant bacterial components in the potato rhizosphere soil at the phylum level revealed a diverse microbial community, with more than 10 phyla being present in both the CK and the four treatment groups. The predominant phyla, including Proteobacteria, Gemmatimonadota, Acidobacteria, Actinobacteria, Chloroflexi, Bacteroidota, Myxococcota, and Verrucomicrobiota accounted for over 90% of the total bacteria (**Figure 4D**).

PCoA showed that there were significant differences in the microbial communities of the five treatment groups (PERMANOVA: R^2^ = 0.763, P = 0.001; **Figure 4E**). The microbial co-occurrence network analysis was conducted at the treatment level during different stages (**Supplementary Figure S1**). The average degree and graph density of the co-occurrence network in the seedling stage treatment were both higher than those of other treatment groups, indicating that the degree of interaction, complexity, and aggregation of rhizosphere microorganisms in the seedling stage treatment were all higher.

### 3.4 Analysis of key bacterial microbiome enriched in the rhizosphere of potatoes treated with nano-selenium

**Figure 5:**
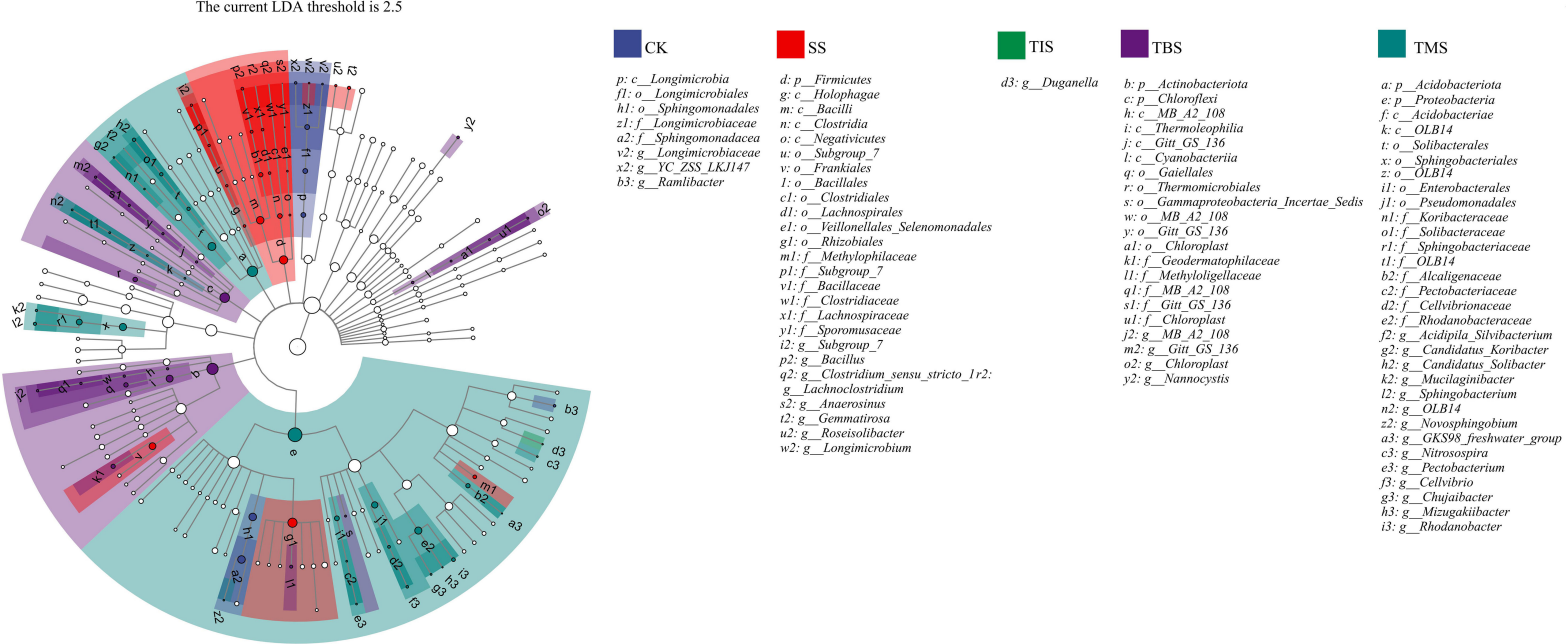
Lefse analysis of biomarker bacteria in the rhizosphere soil after spraying nano-selenium twice at different growth stages. The node size corresponds to the average relative abundance of the taxon; Hollow nodes represent taxons with no significant difference between groups, while nodes with other colors (such as green and red) indicate that these taxons reflect significant differences between groups, and have higher abundance in the grouped samples represented by this color. Letters identify the names of taxons with significant differences between groups. SS, TIS, TBS and TMS were seedling stage, tuber initiation stage, tuber bulking stage and tuber maturity stage, respectively.

Lefse was used to analyze the biomarker bacteria in the potato rhizosphere soil after nano-selenium spraying. The species evolution branching diagram provides a clear understanding of the phylogenetic relationship among microorganisms. At the phylum level, Firmicutes was the biomarker bacteria at the seedling stage; Actinobacteriota and Chloroflexi were the biomarker bacteria at the tuber initiation stage; Acidobacteria and Proteobacteria were the biomarker bacteria at the tuber maturity stage. At the genus level, *Subgroups_7*, *Bacillus*, and *Clostridium* were the biomarker bacteria at the seedling stage; *Duganella* was biomarker bacteria at the tuber formation; MB-A2-108, *Chloroplast*, and *Nannocyitis* were biomarker bacteria at the tuber bulking stage; *Mucilaginibacter*, *Novosphingobium*, and *Pectobacterium* were biomarker bacteria at the tuber maturity stage (**Figure 5**).

**Figure 6:**
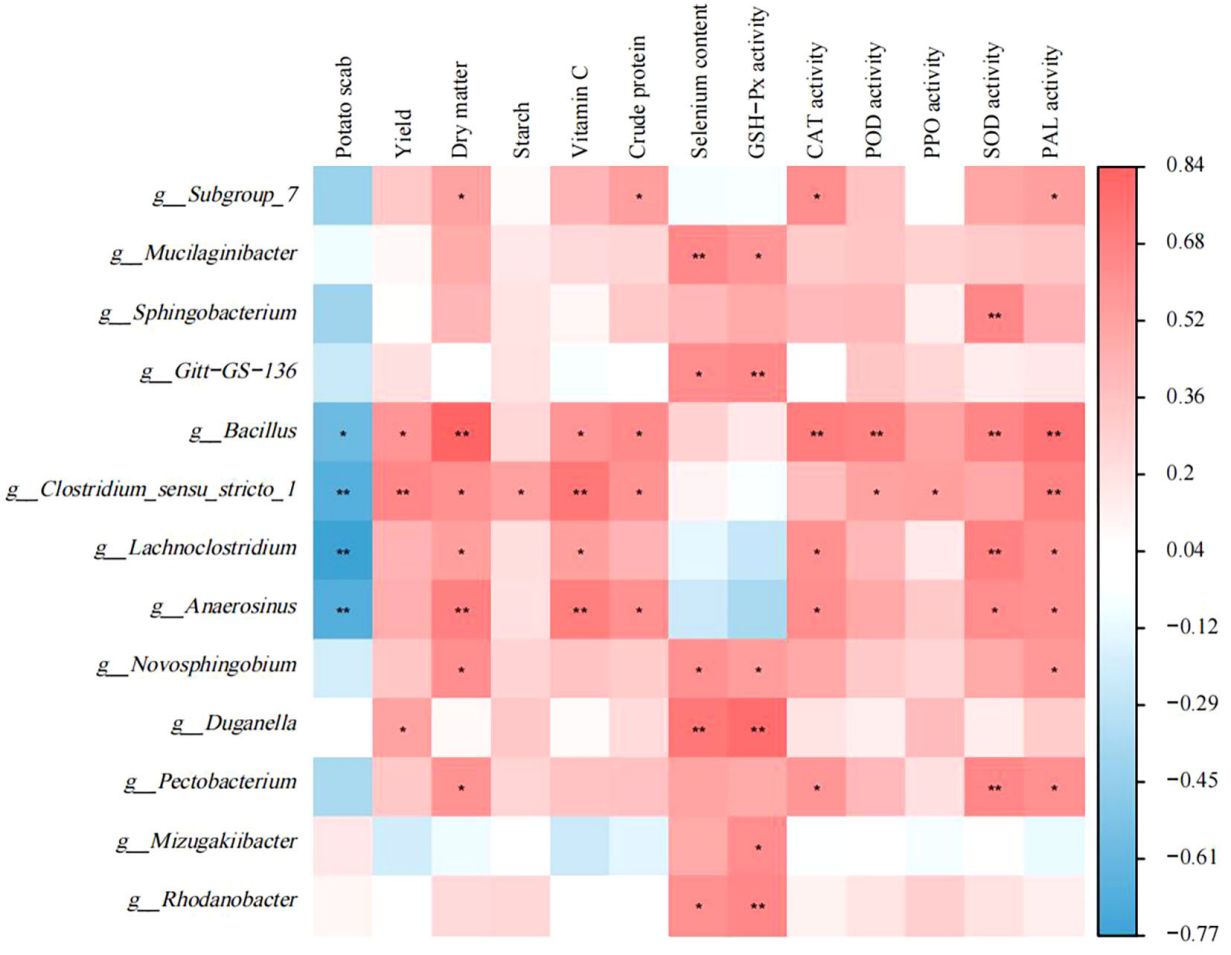
Correlation analysis of biomarker bacteria with disease index of potato scab, yield, potato tuber quality and antioxidant enzyme activity. Red represents positive correlation and blue represents negative correlation. *P< 0.05, **P< 0.01.

Correlation analysis was performed to investigate the relationship between excavated differential microorganisms and disease index of potato scab, yield, potato tuber quality, as well as antioxidant enzyme activity (**Figure 6**). Thirteen bacterial genera exhibited significant correlations with tuber quality. Additionally, five bacterial genera showed significant correlations with the disease index of potato scab. *Bacillus* demonstrated a significantly positive correlation with yield. Moreover, eight bacterial genera displayed significant correlations with antioxidant enzyme activity. The relative abundances of thirteen bacterial genera were significantly correlated with tuber quality. The relative abundances of five bacterial genera were significantly correlated with the disease index of potato scab. The relative abundance of bacteria of the genus *Bacillus* was significantly positively correlated with potato yield. Furthermore, the relative abundances of eight bacterial genera were significantly correlated with antioxidant enzyme activities of tubers.

## 4 Discussion

### 4.1 Effects of nano-selenium on the microbial community in the potato rhizosphere

Foliar spraying of selenium fertilizer can lead to changes in rhizosphere microorganisms (Wang et al., 2024), and this study also found such changes. The foliar spraying of nano-selenium fertilizer can cause changes in rhizosphere metabolites (Wang et al., 2024). The rhizosphere metabolites determine the types and activities of rhizosphere microorganisms (Fitzpatrick et al., 2018). Previous studies have shown that nano-selenium directly enhances the synthesis and secretion of malic acid and citric acid by up-regulating the biosynthesis and transporter protein genes of malic acid and citric acid in rice roots, and subsequently recruits growth-promoting bacteria of the genus *Bacillus* and *Pseudomonas* (Jiao et al., 2023). Furthermore, some of the nano-selenium still infiltrated into the soil through the roots. Existing studies have shown that nano-selenium can induce chemotaxis and biofilm formation of bacteria of the genus *Bacillus* in a dose-dependent manner and promote the colonization of bacteria of the genus *Bacillus* in the rhizosphere (Sun et al., 2024). Selenium enhances the health of plants by optimizing micronutrient levels and stimulating microbial activity (Moulick et al., 2024). From this, we speculate that after the nano-selenium spraying treatment, the components of potato root exudates were changed, thereby affecting the composition of rhizosphere microorganisms. This hypothesis needs to be further verified in the future.

### 4.2 Effects of nano-selenium on potato scab

The health of crops is determined by the diversity of the microbial community. For example, the bacterial Alpha diversity of tomato leaves sprayed with sodium selenite was significantly higher than that of CK, and significantly reduced leaf infection by *Botrytis cinerea* (Li et al., 2022). In this study, it was observed that the Chao1, Shannon and Simpson indices of rhizosphere microorganisms after nano-selenium fertilizer spraying treatment at the seedling stage were significantly higher than those of CK. Furthermore, in this study, the nano-selenium treatment at the seedling stage increased the complexity of the rhizosphere bacterial network. The complexity of the rhizosphere bacterial network is closely associated with disease resistance. For example, in soils with higher bacterial diversity and the more complex symbiotic networks, the key gene abundance and incidence rate of potato scab tended to be lower (Shi et al., 2019).

A large number of studies have indicated that rhizosphere microorganisms are an important component of plant disease resistance. Beneficial microorganisms colonizing in the rhizosphere can directly secrete antibacterial substances to inhibit the growth of pathogens (Karuppiah et al., 2022). For example, studies have shown that bacteria of the genus *Bacillus* inhibit the growth and sporulation of *Streptomyces scabiei* by secreting surfactin, iturin A, and fengycin (Lin et al., 2018). Besides, beneficial microorganisms can induce plants to enhance the activities of antioxidant enzymes such as PAL and POD, thereby resisting the invasion of pathogens (Liu et al., 2024). In this study, through Lefse analysis of the core microorganisms in the rhizosphere soil treated with nano-selenium, it was discovered that bacteria of the genus *Bacillus* were significantly enriched at the seedling stage. The outstanding performance of bacteria of the genus *Bacillus* in controlling plant diseases has been widely reported (Dong et al., 2023; Liu et al., 2024).

### 4.3 Effect of nano-selenium on potato quality and yield

Current studies have shown that the application of selenium could increase the yield and quality of various crops such as maize, rice, and soybeans (Moulick et al., 2024). This study revealed that the content of dry matter, Vc, and crude protein in potato tubers exhibit varying degrees of increase following application of nano-selenium. Additionally, there was a significant increase in the enzyme activities of GSH-Px, CAT, POD, PPO, SOD, and PAL. The small size of nano-selenium makes it to be more easily absorbed and transported by plant cells. After nano-selenium enters the cells, it can act as a selenium source and participate in the synthesis of selenocysteine (Secys), the active center of GSH-Px (Gupta and Gupta, 2017). Numerous studies have demonstrated that selenium enhances the activity of antioxidant enzymes such as GSH-Px, CAT, and POD in crops, thereby safeguarding cell membranes against oxidation and damage caused by peroxides and free radicals, helping to maintain the structural and functional integrity of cell membranes (Çatav et al., 2022; Feng and Wei, 2012; Li et al., 2021). Enhancing the antioxidant capacity of tubers is one of the reasons why nano-selenium can improve the quality of tubers. Previous studies have shown that nano-selenium spray on celery induces the biosynthesis of flavonoids and phenolic substances by stimulating the leaf linolenic acid pathway, and enhances its antioxidant capacity (Li et al., 2020). Moreover, some phenolic compounds also have the activity of inhibiting pathogens, which can further reduce the adverse effects of diseases on crop quality (Wu et al., 2024).

On the other hand, adequate supply of nutrients is another reason why nano-selenium can improve the quality of tubers. When selenium fertilizer is sprayed, the beneficial microorganisms enriched in the rhizosphere can help potatoes absorb selenium and other important nutrients, such as nitrogen, phosphorus, and potassium, more effectively. The sufficient supply of these nutrients is crucial for the growth and quality improvement of potato tubers. Some beneficial microorganisms, such as bacteria of the genus *Bacillus* can secrete various proteases and ureases to decompose a large number of organic nitrogen compounds existing in the soil (Huang et al., 2019; Mo et al., 2024). They convert organic nitrogen into inorganic nitrogen forms, enabling crops to absorb nitrogen more effectively. Nitrogen is a component of important biological macromolecules such as proteins and chlorophyll in plants (Sawicka et al., 2020). More nitrogen absorption helps increase the protein content in crops, which is consistent with the significant increase in dry matter and crude protein content of tubers after spraying nano-selenium fertilizer in this study. Additionally, some beneficial microorganisms can also secrete substances such as organic acids. These substances can dissolve the insoluble nutrients in the soil and transform them into forms that can be absorbed by potatoes. For instance, bacteria of the genus *Bacillus* can secrete organic acids to promote the dissolution of phosphorus in the soil and enhance the availability of phosphorus (Liu et al., 2024). The existence of beneficial microorganisms can further enhance the absorption of nutrients by potatoes, providing a sufficient nutritional basis for the improvement of tuber quality and yield. Studies demonstrated that selenium exerted a positive influence on the yield of numerous crops (He et al., 2019; Jiang et al., 2017; Zhang et al., 2019). Application of selenium through foliar spraying could enhance the chlorophyll content in leaves, stimulate photosynthesis, facilitate plant growth, and consequently augment crop productivity (Lai et al., 2019). Furthermore, the results of this study indicate that by enhancing the activity of antioxidant enzymes in plants, promoting the transformation or accumulation of various nutrients in tubers, and reducing the occurrence degree of potato scab, it is also an important reason why nano-selenium can increase yield.

### 4.4 Exploration of the optimal spray frequency and period of nano-selenium

The different spraying frequencies of selenium fertilizer have varying effects on different crops (Gu et al., 2024; Huang et al., 2023). Therefore, in this study, the effects of different spraying frequencies on tuber quality were comprehensively compared. In the spraying twice treatment, MFVQ index of potato tuber was the highest. This may be due to the existence of a relatively optimal range of selenium content that affects tuber indexes, where the best effects on these indexes are achieved within this range. Therefore, nano-selenium should be sprayed twice in potato agricultural production to reduce the cost.

The foliar application of selenium is an effective method for enhancing the selenium content in fruits. The appropriate stage for selenium spraying varies among different crops. For instance, when treated with selenate, rice grains at the full panicle stage exhibited significantly higher selenium levels compared to those at the late tillering stage (Deng et al., 2017). Under the foliar spraying of selenite and selenate during the tuber expansion stage, the selenium content of potato tubers was significantly higher than that in the tuber formation stage and tuber maturation stage (Zhang et al., 2019). This finding is different from our research results. That is, under the foliar spraying of nano-selenium during the tuber formation stage, the selenium content of tubers was significantly higher than that in other period treatments. The possible reason behind this result is that during the tuber initiation stage, plants will allocate a large amount of nutrients (including selenium) to the forming parts of new tubers.

Compared to inorganic selenium, nano-selenium has smaller particles and a higher specific surface area, which makes it easier to be absorbed on the leaves (Wang et al., 2020). After nano-selenium fertilizer is sprayed onto the leaves, it can be smoothly transported to the tubers through the active nutrient transport channels in the plants, such as the phloem (Xia et al., 2020). Studies have shown that exogenous nano-selenium has a sustained-release effect in the soil, can promote the growth of soil microorganisms, enrich soil probiotics, and has low toxicity and high safety to the soil environment (Liu et al., 2021). Therefore, it is safer and more reliable for application in agriculture. However, the influence of nano-selenium on crops is also restricted by various factors. In practical applications, depending on different crops, the application period and dosage of nano-selenium should be reasonably controlled to effectively increase crop yield and quality.

## 5 Conclusions

In conclusion, the results demonstrated that the application of nano-selenium twice during the seedling stage exhibited superior efficacy, significantly enhancing potato scab resistance, yield, tuber quality, and antioxidant enzyme activity. The diversity index of the rhizosphere bacterial community increased significantly, and at the same time, the complexity of the symbiotic network also increased. Bacteria of the genus *Bacillus* and other beneficial microorganisms were enriched, and their relative abundance were significantly correlated with tuber quality, yield, disease severity index, and antioxidant enzyme activity. The present study offers theoretical support for the investigation of a potato selenium-enriched technology system and provides scientific guidance for the utilization of nano-selenium.

## Data Availability

The original contributions presented in the study are included in the article/**Supplementary Material**. Further inquiries can be directed to the corresponding authors. The high-throughput sequencing data presented in the study are deposited in the NCBI, accession number SRR32079287-SRR32079304.

## Supplementary material

Supplementary Figure 1 Genus-level microbial co-occurrence networks of rhizosphere soil bacteria. Nodes indicate bacterial genera with relative abundance in the top 100, and edges represent significant co-occurrence relationships (Spearman’s ρ > 0.8 and P< 0.05). SS, TIS, TBS and TMS were seedling stage, tuber initiation stage, tuber bulking stage and tuber maturity stage, respectively.
