# Supplementary figures and images for: Nano-selenium strengthens potato resistance to potato scab induced by Streptomyces spp., increases yield, and elevates tuber quality by influencing rhizosphere microbiomes
Source: Front Plant Sci. 2025 Feb 3;16:1523174. doi: 10.3389/fpls.2025.1523174 (PMC11830815; doi:10.3389/fpls.2025.1523174)

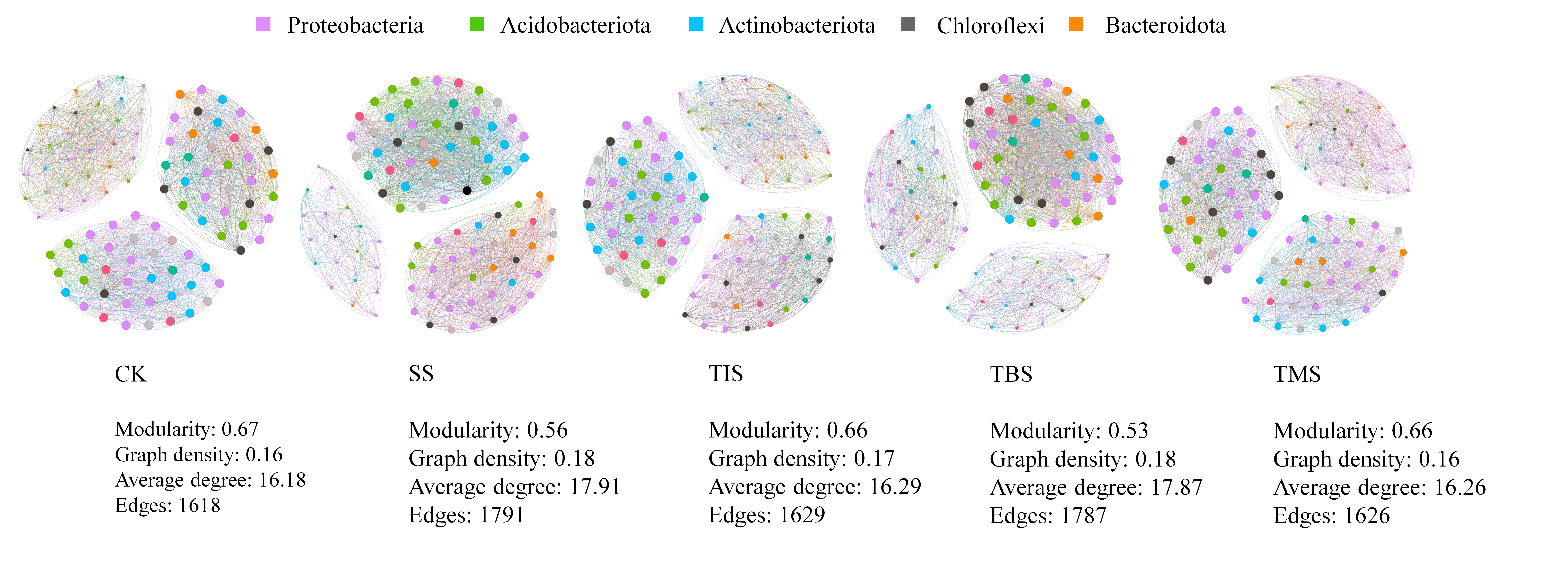

Supplement: Supplementary Figure 1 — Genus-level microbial co-occurrence networks of rhizosphere soil bacteria. Nodes indicate bacterial genera with relative abundance in the top 100, and edges represent significant co-occurrence relationships (Spearman’s ρ > 0.8 and P< 0.05). SS, TIS, TBS and TMS were seedling stage, tuber initiation stage, tuber bulking stage and tuber maturity stage, respectively. [file Image1.jpeg]
